# Supplementary material for: Patterns of Intron Gain and Loss in Fungi
Source: PLoS Biol. 2004 Nov 30;2(12):e422. doi: 10.1371/journal.pbio.0020422 (PMC532390; doi:10.1371/journal.pbio.0020422)
Supplement: Table S1 — Also available at http://genes.mit.edu/NielsenEtAl/. (4.3 MB ZIP). [file pbio.0020422.st001.zip › NielsenEtAl/html/361.html]

AN1800.1.NCU04615.1.MG07312.1.FG10042.1


```
 CLUSTAL W (1.82) Multiple Sequence Alignments - Introns Inserted


Sequence 1: NCU04615.1	1266 aa
Sequence 2: FG10042.1	1148 aa
Sequence 3: MG07312.1	1206 aa
Sequence 4: AN1800.1	1080 aa
Alignment Length: 1298 aa
Number Identitical Residues: 400 aa
Alignment Score (without introns) 20321


MG07312.1 	MRIAIREQLALLVVLATLVALAIVSIPV~WIYVHQFIIGVQ~ENALSLTASLKAARIAAE
NCU04615.1	MRIAIREQLAALVIFAVLLSLAIVSIPV~WIFVNNFVIGVK~TDGLALSASLKASRISSE
FG10042.1 	MRIAIREQLAALVLLAVLVSLAIVSIPT~WIYVNRFVVDIE~RDGLQLTASLKASRISAE
AN1800.1  	MRVPIAVQLGLLVLLTALAGLAALAIAT0WINNYNFVVDVK2SQSLQLVASIKSSQITSN
          	**:.*  **. **:::.* .** ::*.. **   .*::.::  :.* * **:*:::*:::

MG07312.1 	IDLLQTTCSTIASRVLVQRALLNFYN----GSSSPSLWDEARIDVENALNLPGQPDLLQA
NCU04615.1	IDLIQTACTTISTRLLLQQAFEDFYTNEYYGNQTADVWEQALEDLASALSTSNVSGLLQA
FG10042.1 	LDLIETIAMTISSRILVQNALTNYYN------TGKNEWEDAHEDLESALSVSTASGLLQI
AN1800.1  	LDLLETTSKTIITRILIQSALQRYYAG----NTTEENFASSITDVQSALGSRGYLSLYQA
          	:**::* . ** :*:*:* *:  :* .    .   . : .:  *: .**.     .* * 

MG07312.1 	RIYSRNSTG--SSSTGLMATTSTMFH--DIKLPYNKPDGTSAMLGNDSYGFPPMLYPNIT
NCU04615.1	RLYSRNTTG--DPRG-LLNITGQDALDDPVYLPYRSPTGADIKLGDPDYGFPPMLYPNIT
FG10042.1 	RLFSRNNTK--TKGGVLLNVTSLSVP--EIELPYKDSEGSNVRLGDTDLGYPPSLFPNLT
AN1800.1  	SIYARNVENGESKARRLLNVTSDEVP--EIRLPYDHPNGTAVMLGDEGLGYPPSLYPNLT
          	 :::**   ..     *:  *.       : ***  . *:   **: . *:** *:**:*

MG07312.1 	YTDTGEPSPIAPDTNIFFADAFPGIRLTQ-----GLFLGPLVINDTYALVSLTVAVRGNA
NCU04615.1	YENKNLPSPYKANTVQFSANIFPGVSLSNGSEGNGILLGPLVVNESYALVSITVPVRENQ
FG10042.1 	YIDLERPNNVRNNTTAYAAEAFDDVRIARDG---GLLLGPLVLNESASLLSVTIPVRDNN
AN1800.1  	-----------EDATTNKLYAFDGVPITTNQP---LLLGPLATNDSFSLISLSLPIINNT
          	            ::       * .: :: .     ::****. *:: :*:*:::.:  * 

MG07312.1 	DK-LILGYMT~LVASASNLILIRNSPEGLGNSGLTLLFGPNETYNRFNYTDPPVTNSTYK
NCU04615.1	SSRFILGYMT~VIASARQLVQVQRSNEGLGETGVALFIGPVNPWNHFNSKMP-ASNQTWS
FG10042.1 	DT-FILGYMS~VVTLAQSLTAIQDSREGLGETGSVLLIGPTDRSNRFNKTQP-ASNATYT
AN1800.1  	SAADILGYIT2VVASAANLQDAMSSRQGLGSSGQVLLLAPSRPENRFPTGSRPATATSE-
          	.   ****:: ::: * .*     * :***.:* .*::.*    *:*     ..:  :  

MG07312.1 	IPDTFKFAKTKIHFVLPPTKPNGTADRHLKHSFDSGNYNEEFELGSYPAVFESLTTFNRR
NCU04615.1	PPRDEFARTNQVHFLLPPAPQPGQEDRHSDHSFESGAYDAPFDPATYQAVLDLFVEKNN-
FG10042.1 	PNRDEFG-NVMVKFILPPKSPEGHKDLHDVHQFVSEYGNRAFPVKNYPAALDSFTKHYN-
AN1800.1  	-PQIAGLDNQEVRYVFRPTPLPGNKRRHSP-AVASG---ASFPLRDYPIALKVLNQPYD-
          	        .  ::::: *    *    *    . *      *    *  .:. :      

MG07312.1 	AVNNAMANLWTTNEQGVDVSVGTARPKSQYVDWVVVVEQDRAEALQAITTLRTILLGCVF
NCU04615.1	QTNNATSKISTKNEQGKKVAIGAARPQTSLVTWAVIVEQDKREATEPIRTLRNILLGCVF
FG10042.1 	TVSNASSLINTHNEQNARVAVGFARTQTPLANWTVIVEKAKWEAYKPINTLRNILLGTVF
AN1800.1  	SINHSITDLSSKNENGTRVAVGAIQQRSDLVEWVLIIEETHGEAFAPVSRLRKIILACVF
          	  .:: : : : **:.  *::*  : ::  . *.:::*: : **  .:  **.*:*. **

MG07312.1 	GAAGLMFLLIFPCAHFSVRPIRDLKIATEKSIMPPGYDD-----YHGGTSENPGSGVLSS
NCU04615.1	GTAGLVLLLTIPCAHLSVMPIRRLKEATEKSIAPPGYED---LSDSDYDEENPSSGGTTS
FG10042.1 	GTVGLIALIIVPCAHVSVLPIRKLKAATEKSVNPPGYEDSFIDSDSDFDDEIPSSGGQSR
AN1800.1  	GTAGVIALLIPPLAHLSVAPIRRLREATRKSVNPTIGPLS-----PSLSTMVDPIEISMH
          	*:.*:: *:  * **.** *** *: **.**: *.    :      .             

MG07312.1 	-LSEKGWLGAIIRRTRHKLGIPARRQRRESDQDESLTQRQVFKIPGKVQDRKHYITDELT
NCU04615.1	GRSEKSWLKSLKRRMR---KSKRARLRAAAAQD---AHRHAFKIPAKVEDRKHYITDELT
FG10042.1 	RSKKKGPISKFIRRLK---GKSNG---PSSNRD---VARRMFKIPAKVDDRKHFVTDELT
AN1800.1  	NGNEKSEKGGFFNRLK----RGRGNLATGHDRD---NEPHQFQIPGRVKERKHIITDELT
          	  .:*.    : .* :               :*      : *:**.:*.:*** :*****

MG07312.1 	ELTTTFNEMTDELYKQYSSLDDKVAERTRELEISKKAAEAANESKTLFIANISHELKTPL
NCU04615.1	ELTQTFNEMSDELLKQYTSLDEKVAERTRELEISKKAAEAANESKTLFIANISHELKTPL
FG10042.1 	ELTRTFNDMSDELVKQYLSLEVKVAERTRELEESKKAAEAANESKTLFIANISHELKTPL
AN1800.1  	DLTKTFNEMSDELVIQYNRLEERVAERTRELEKAKIAAETANESKTLFIANISHELKTPL
          	:** ***:*:***  **  *: :********* :* ***:********************

MG07312.1 	NGIMGMCAVCMEETDVTRIKQSLKTLYKSG~DLLLHLLEDLLSFSKNQIGHQISLEERDF
NCU04615.1	NGIMGMCAVLMEEDDVLRIKQSLKTIYKSG1DLLLHLLEDLLSFSKNQIGQQVSLEEREF
FG10042.1 	NGILGMCAVCMEENDIVRIKQSLKTLYKSG~DLLLHLLEDLLSFSKNQIGQQLSLELREF
AN1800.1  	NGILGMCAVCMGEDDLPRIKRSLQTVYKSG~DLLLHLLNDLLTFSRNQIDQAIRLEEKEF
          	***:***** * * *: ***:**:*:**** *******:***:**:***.: : ** ::*

MG07312.1 	RLGDIRQQLLSIFDKQVRESGTHFTVEFVGHDILDFYQYPISSERQSMEKTRLPALGPAG
NCU04615.1	RLGEIRSQMLAIFDKQIRENKITFTVSFVGTDILES----SPE-RRSGIDKRLPALGPPG
FG10042.1 	RLADIRSQILTIFDKQVREGKINFSVDFISSDNAEF----SASPDRTNGETRLPALGPHG
AN1800.1  	KLSDIRSQLQIIFQNQVQEKQISFSIKFCHPDTPPIN-----DTPEVISRAPGPVYGPPG
          	:*.:**.*:  **::*::*    *::.*   *          .  .       *. ** *

MG07312.1 	AGRLKDMCLWGDQHRILQVIINLVSNSLKFTPSGGKVQLRIRCVEEIEQINATGDNSRSS
NCU04615.1	VGRLKDMCLWGDQHRILQVIINLVSNSLKFTPAGGRVELRIRCVGEMEQTH-NQSRSSSL
FG10042.1 	TGRLKDMCLWGDQHRILQVIINLVSNSLKFTPHNGTVSVRIRCLGEAEALAENDSRASSI
AN1800.1  	TAKLKDMVLWGDQHRILQILINLVGNSLKFTPEQGTVAIRIKCVGE----------EKNP
          	..:**** **********::****.*******  * * :**:*: *            . 

MG07312.1 	SFSKSGSARPGRPRHRVGSSSAQSAASTKGVSTPG-TTGTALAINPMDPKTVPHIQVRER
NCU04615.1	SKNSHRPGRS-RHRLGSGSRDSQSSKGAPHSPNLNQSSGTALSINPMDPKS-PHVHIRER
FG10042.1 	SKNSRNNSRTGRPRQRGSASSANSGASGRNSAQKFVSTGTALSINPRDPKATPHVQVRER
AN1800.1  	SASPRPESRALSRWRSIGSSHASSRPK------------TSLDR-PRDGGD---------
          	* .    .*.       .:  :.*               *:*   * *            

MG07312.1 	SLSPPPPGAKAFLFEFEVEDSGPGIPEHMQARVFEPFVQGDLGLSKKYGGTGLGLSICQQ
NCU04615.1	SPTPPPPNAKTFMFEFEVEDTGPGIAEHMQQKIFEPFVQGDLGLSKKFGGTGLGLSICSQ
FG10042.1 	SPTPPPPGAKSYIFEFEVQDTGPGIPEHMQQKVFEPFVQGDLGLSKKFGGTGLGLSICHQ
AN1800.1  	EETTRPPNLRTLILQFEVEDTGPGIPQHLQNRVFEPFVQGDLRLNRKYGGTGLGLSICSQ
          	. :. **. :: :::***:*:****.:*:* ::********* *.:*:********** *

MG07312.1 	LATLMGGNITLRSTVGVGSVFTMRIPLKYVKDR2QQTIRPSSTASSSIKSRPQSLVSTDG
NCU04615.1	LATIMGGTIGLKSTVGVGTTFTMRIPLKYVRDR2-----ASSTASSSLASRPPSAGSLDG
FG10042.1 	LAGLMGGSIKLRSSVGVGTTFTMQIPLKYVKDR2-----TSSTASSSIKSRPTSVDAGD-
AN1800.1  	LARLMRGQIQLNSEQGRGTLFLVRIPLGFVKES~PPS--TASSMTSLAGSRTSSVFSLDD
          	** :* * * *.*  * *: * ::*** :*::    :  .:*: :*   **. *  : *.

MG07312.1 	-----------DTTNVRVVSH-----------SKSAPDGKHASVLDNKPRLVGLSHPFFS
NCU04615.1	ETIRNPLPKQSTIDIHRSHSHGHSHSHNHGHDQHSTAPPAAASVLDTQPRLVGLSQPFFA
FG10042.1 	------------LELQRTAPS----------TPKPAAEAKSAGALNPQPRLVGLSQPYFA
AN1800.1  	-----------LASVTKAPSN-------HSVASQHESTKSNAEIQDSQPRLVGLSQPFFA
          	                :  .         .   :  .    *   : :*******:*:*:

MG07312.1 	PTQATAPKLSTEDHKANLNEKIAKAVAAK---GGNKLRVLVADDNSTNVEVVSKMLRLEH
NCU04615.1	TTPTTSG--DVDDKMAAIDRAMAAKQLQQSSSGPGKLRVLVADDNSTNVEVVSRLLKLEE
FG10042.1 	ANPPAVK--STQQKMAEIDRAMANKSGQ-------RVRVLVADDNSTNIEVVSRMLKLED
AN1800.1  	PPVPQAK----DNGLKKIRALVAEDNVVN----QEVVLRYALLFRFVSCTDPCRMLALED
          	.  .       ::    :   :*     .       :   .   . ..    .::* **.

MG07312.1 	VSDVTVASDGQEALEVVKANMENNLYFDLIFMDVQ~MPNLDGLQSTRLIRQMGYSAPIVA
NCU04615.1	VYDVTIAKDGQEAYDLVKANMEHNLQFDVIFMDIQ~MPNLDGLQSTRLIRQMGYSAPIVA
FG10042.1 	VYDVTIAKDGQEAYDLVKATMEKNQSFDVIFMDVQ~MPNVDGLQSTRLIREMGYKSPIVA
AN1800.1  	VYDVTVVKDGQEAYDTVKANMEEGKVFDLIFMDIQ0MPNLDGIESTRLIRQMGCSAPIVA
          	* ***:..***** : ***.**..  **:****:* ***:**::******:** .:****

MG07312.1 	LTAFSEESNVKECMESGMNEFLA~KPIRRPALKQVLRKFATIPEEPEDGRRTTPPTSAKN
NCU04615.1	LTAFSEESNVKECMESGMDEFLS~KPIRRPALKQVLKKFATIYEEQGETETVPSVTTGS-
FG10042.1 	LTAFSEESNVKECIDSGMDEFLA~KPIRRPALKQVLKKFATIPEEP--------------
AN1800.1  	LSAFSEDSNIKDCMNSGMDMFIS2KPIRRPALKQVLRRFTPIPEEAE-------------
          	*:****:**:*:*::***: *:: ************::*:.* **               

MG07312.1 	VVTDSHGTKSSVRTEVAETKREDLPNGNGNAVVGPDNNSSSVLISKP-------------
NCU04615.1	--MKGEKKDEKVEKKDKKEGREWKGMKDGKGDGSLVNGTITPKTASSTTVAATMTNGSAM
FG10042.1 	---ENEKRSSSLKPKARANGHAVPPHKEGE------------------------------
AN1800.1  	------------KPQM--------------------------------------------
          	            . :                                             

MG07312.1 	--PLPPMNGAAVAT-------------------------------
NCU04615.1	TGPTTMMNGAPMRPKPVDVYSDEPSPKTMSKSDGAAKGDYLKRGR
FG10042.1 	-------NGAPKE----------------------------KHG-
AN1800.1  	---------------------------------------------
          	
```
